# Supplementary material for: Are carbon emissions trading and green financial instruments synergistic? -Comprehensive quantitative research based on content analysis
Source: PLoS One. 2024 Mar 7;19(3):e0298601. doi: 10.1371/journal.pone.0298601 (PMC10919601; doi:10.1371/journal.pone.0298601)
Supplement: S1 Data — (ZIP) [file pone.0298601.s001.zip › Data/地级市Z_F工作报告.zip!/─■╧─/╬Γ╓╥╩╨/└┤╫╘╣1⁄2╓┌║┼í╛┬φ┐╦╩2╛▌═°í┐_2018.docx]

各位代表：

现在，我代表市人民政府，向大会报告政府工作，请予审议，并请市政协委员和其他列席人员提出意见。

2017年工作回顾

2017年是极不平凡、具有里程碑意义的一年。党的十九大胜利召开，确立了习近平新时代中国特色社会主义思想的指导地位，描绘了建设社会主义现代化强国的宏伟蓝图。自治区第十二次党代会提出的“三大战略”和“五个扎实推进”，明确了全区的奋斗目标和主要任务，增强了我们实现经济繁荣、民族团结、环境优美、人民富裕，与全国全区同步全面建成小康社会的信心和决心。

一年来，在自治区党委、政府和市委的坚强领导下，在市人大、政协的监督支持下，全市上下以习近平新时代中国特色社会主义思想为指导，深入学习贯彻党的十九大精神，按照自治区第十二次党代会的部署要求，全力做好稳增长、促改革、调结构、惠民生、防风险各项工作，保持了经济社会持续健康发展的良好势头，全面建成小康社会迈出新的步伐。全年实现地区生产总值508.1亿元，增长8%；全社会固定资产投资798.8亿元，增长8.2%；一般公共预算收入32.7亿元，同口径增长10%；一般公共预算支出200.1亿元，增长10.2%；社会消费品零售总额112.7亿元，增长9.7%；城镇居民人均可支配收入25364元，增长8.6%，农村居民人均可支配收入突破万元大关，达到10912元，增长9.8%。

坚持精准施策，经济发展平稳向好。深入实施工业转型升级、创新驱动、消费拉动经济增长等19项行动计划，兑现各类奖补资金2亿元，争取自治区“降成本30条”惠企政策资金2.4亿元。包抓中国自动化产业基地、大坝电厂四期等重大项目，200个市级重点项目开工197个，完成投资462亿元。包扶可可美生物公司等25家重点企业，解决各类困难问题75个。实施PPP项目12个，总投资33亿元，撬动社会资本30亿元，我市被国家发改委、住建部列为重大市政工程领域PPP创新工作重点城市。设立“中国制造2025”等8支产业基金，规模达到23亿元。抢抓机遇招商引资，全国工商联常委会议签约项目101个，投资972亿元，中阿博览会签约项目16个，投资347.6亿元。全年实施招商项目316个，到位资金658亿元。争取项目1250个，落实资金138.2亿元。

着力调整结构，质量效益全面提升。工业转型步伐加快。全力推进“中国制造2025”试点示范城市创建，新疆特变电工新能源配套装备制造等项目加快推进，汇高科技等50个工业项目建成投产。装备制造业实现产值40亿元。纺纱产能达到70万锭。风光电建成并网发电706.6万千瓦，占全区48.1%。实施勤昌轴承自动化生产线等技术改造项目15个，淘汰水泥、造纸等落后产能69.1万吨。太阳镁业等3家企业被评为国家两化融合贯标试点企业。落实低成本化园区改造项目23个，完成投资6.6亿元。新增规上企业55户，规上工业增加值增长10.4%。万元GDP能耗下降1.5%，单位工业增加值能耗下降7%。现代农业稳步发展。粮食产量达到97.7万吨，冬麦后复种水稻“一年两熟制”试点成功。新增瓜菜、枸杞、黄花等特色种植20.8万亩。奶牛存栏22.1万头，养殖规模化率达到96%。肉牛、肉羊饲养量分别达到43万头、640万只。富硒农业种植面积达到11万亩。组建亚麻籽油等产业联盟。新增农业产业化龙头企业35家、家庭农场105家、合作社93家。下马关等5座中部干旱带水库建成运行。利通区被评为国家农产品质量安全县，青铜峡市被确定为国家优质粮食工程试点县和现代农业可持续发展试验示范区，吴忠国家农业科技园区被认定为国家有机食品生产基地建设示范区。“盐池滩羊”进入首批中国特色农产品优势区名单，塞外香等5个品牌被评为宁夏特色优质农产品品牌。金沙湾、汇达、红粉佳荣、贺兰芳华4家酒庄被评为自治区五级列级酒庄，维加妮等11家酒庄的15款葡萄酒获得国际葡萄酒大赛金奖。第三产业加快发展。万达广场加快建设，茶茗文化街、光耀小吃街等特色街区建成运营。成功获批创建国家电子商务示范市，电商交易额达到11.5亿元。新建改建养老服务机构12个，全区首家公建民营养老服务机构投入运营。房地产销售面积163.8万平方米，增长8.4%，库存下降25.8%。成功举办第十届国际葡萄与葡萄酒学术研讨会、盐池航空嘉年华、红寺堡全国青少年航空航天模型锦标赛等节会活动。上桥镇牛家坊村、叶盛镇被评为第七批全国一村一品示范村镇。中华黄河楼成功创建为国家4A级景区。全年接待游客675.3万人，实现旅游收入38.15亿元，同比分别增长20%、30%。

注重统筹协调，城乡建设同步推进。实施城乡建设项目164个，完成投资143亿元。加快利青同城步伐，积极融入银川都市圈建设。全力配合银西高铁、吴忠至中卫城际铁路和京藏高速改扩建3个重大项目建设，青铜峡至银川正源街快速通道、国道344线吴忠至灵武段公路开工建设。加快黄河文体会展中心建设，建成地下综合管廊6.2公里，市区热电联产集中供热面积达到1436万平方米，综合改造老旧小区60个。建成城市道路52公里、农村公路480公里。实施棚户区改造13195套，建设美丽小城镇6个，美丽村庄示范点35个，改造农村危房8596户，农村改厕治污8340户。金银滩镇、韦州镇被列为全国第二批特色小镇。光纤覆盖所有行政村，全国电信普遍服务试点工作现场交流会在我市召开。城镇新建绿色建筑比重达到30%，街道清扫机械化作业覆盖面达到85%以上。实施农村环境综合治理行动计划，农村人居环境进一步改善。城市更加宜居宜业，农村展现新的气象。

落实生态优先，环境质量持续改善。加快推进生态立市，“三区三线”基本划定。启动国家森林城市创建工作，全市完成营造林30.16万亩，森林覆盖率16.02%，城市建成区绿地率、绿化覆盖率、人均公园绿地面积分别达到40.7%、41.9%和20.1平方米。深入开展“蓝天碧水•绿色城乡”和“绿盾2017”自然保护区清理整治专项行动，中央第八环保督察组反馈问题得到切实整改。加大环保执法力度，实施行政处罚181件。市区集中供热管网覆盖范围内20蒸吨以下燃煤锅炉全部淘汰，淘汰黄标车1655辆、老旧车5206辆，空气质量优良天数达到292天，比上年增加12天。在全区率先推行河长制，市县乡村四级河长实现全覆盖，845名各级河长到位履责。加快推进清水沟、南干沟水环境综合治理，水质基本达到四类标准。完成9个生活污水处理厂、7个工业园区污水处理厂提标改造工程。全力确保黄河水质安全，封堵取缔23家企业排污口，黄河吴忠段保持_x0007_II类优水质。中科国通生活垃圾焚烧发电项目建成运营。划定永久基本农田361万亩、禁养区60个1417平方公里。

加快改革创新，发展活力不断增强。顺利完成空间规划（多规合一）改革试点。在全区率先启动市辖区综合执法改革，组建吴忠市综合执法监督局和利通区、红寺堡区综合执法局。积极推进“不见面、马上办”审批服务，建成太阳山、下马关政务服务中心，12345便民服务中心挂牌运行，群众办事更加便捷。我市“互联网+政务服务”工作经验在全国交流，市公共资源交易中心被评为全国十佳公共资源交易中心。清理规范行政事业性收费25项，获批外商投资企业核准登记权，工商注册便利化受到国务院表扬。新增市场主体7770户，增长8.58%，总数达到98333户，民营经济占比达到51.6%。利通区被列为全国第二批村集体资产股份权能改革试点县。市属国有企业资产总额达到159亿元、实现利润3994万元、完成融资41.78亿元，分别增长25%、2倍和1.6倍。新增驰名商标1件、地理标志证明商标1件，我市荣获全国十大地理标志商标精准扶贫典型市。国投（宁夏）互联网小额贷款公司在吴忠注册成立，嘉泽新能源成为宁夏14年来首家A股上市企业。建成公交车智能调度系统，新购新能源公交车30辆，群众出行更加方便。我市被确定为国家知识产权试点城市，金积工业园区被确定为国家知识产权试点园区。鑫浩源生物科技等3家企业被认定为国家级高新技术企业。新增国家知识产权优势企业3家，自治区科技型中小企业26家、自治区技术创新中心3家、自治区企业“双创”平台2家。吴忠仪表被认定为国家级国际科技合作基地、全国制造业信息化示范企业。佳能创科化工等33家企业被认定为自治区“专精特新”中小企业，夏进制箱包装等27家企业被认定为“专精特新”示范企业。建立吴忠国家农业科技园区蔬菜产业和设施园艺，君星坊公司亚麻籽产品，青铜峡菲斯克汽车轴承4个院士工作站。设立青少年市长创新奖，5个单位、18名个人受到表彰奖励。重庆等20个优质特色产品展示展销中心建成运营，总数达到35个，销售额突破1亿元。进出口总额达到6.46亿元，同比增长84.5%。成功举办第六届宁夏黄河金岸（吴忠）国际马拉松赛、全国沙滩排球巡回赛（吴忠站）等体育赛事。

牢记初心使命，民生得到切实改善。高质量办结10件民生实事。脱贫销号59个村，减少贫困人口48206人，盐池县脱贫摘帽通过自治区先期评估。整合投入扶贫资金62.5亿元，设立各类扶贫产业担保基金6.82亿元，累计发放扶贫小额信贷18.53亿元，建档立卡贫困人口家庭意外伤害保险和大病补充医疗保险免费全覆盖，建档立卡户大病保险起付线由8400元调低到3000元，大病救助上限由8万元调高到16万元。全面完成“十三五”易地搬迁住房建设任务，搬迁1530户6386人。同心县“扶贫车间”经验在全国交流，红寺堡“壹加壹”获中国畜牧产业扶贫优秀模式。发放创业担保贷款1.3亿元、农村妇女创业担保贷款18亿元。新增城镇就业1.4万人，城镇登记失业率3.52%，农村劳动力转移就业21.5万人。城乡低保提标扩面，城乡居民基础养老金标准从每人每月150元提高到170元。残疾人、红十字、慈善等事业健康发展。投资6.5亿元，新建、改扩建学校（幼儿园）102所，营养改善惠及7.1万名学生。职业教育提质扩面，产教融合深入推进。红寺堡区、同心县顺利通过自治区义务教育均衡发展验收。全市高考一二本上线率达到35.97%，中考平均分同比提高22.6分。市人民医院新院全面投入使用，法人治理结构和人事薪酬制度全面建立，与国内17家知名医院、医疗团队和知名专家签订合作协议。全面落实分级诊疗制度，公立医院药品加成全部取消。建成标准化村卫生室47所、社区卫生服务站8所。普通人群、重点人群家庭医生签约服务覆盖率分别达到39.9%和73.1%。成功创建为国家卫生城市、全国幸福家庭活动示范市。盐池县医改工作受到国务院表扬。人口计划生育、妇幼保健、疾病预防等工作得到加强。公共文化和全民健身活动蓬勃开展，新建贫困地区村级综合文化服务中心132个，开展文化惠民演出655场，成功举办吴忠市第四届运动会，红寺堡体育馆、盐池全民健身中心建成运行。国家公共文化服务体系示范区创建工作扎实推进，中期评估位列西部地区前列。引黄古灌区成功列入世界灌溉工程遗产名录，鸽子山遗址入选全国十大考古新发现。推出《锦鸡岭》等一批文艺精品力作。创建全国文明单位5个、文明村镇4个、文明校园1个，朱玉国等3人荣获全国道德模范提名奖。涌现出脱贫攻坚优秀共产党员李进祯等一批先进典型。移风易俗工作被全区观摩学习。新闻出版、广播电视、住房公积金、文史、档案等工作都有新进展。

加强综合治理，社会大局和谐稳定。制定实施《吴忠市宗教事务依法管理全面量化体系》，大力实施民族团结“细胞工程”，深入开展“四进”宗教场所、军警寺共建等特色民族团结创建活动，自治区“双创”工作互观互检现场会在我市召开。生产安全事故起数和死亡人数分别下降16%和6.98%，经验做法两次被国务院安委会肯定推广，我市被国家安监总局确定为全国安全生产领域改革发展联系点。深入开展“七五”普法，“广场说法•一周一法”“以案释法”等法制宣传深入人心。信访批次、人次分别下降18%和22%。市劳动监察大队被评为全国清理整顿人力资源市场先进单位。积极创新人民调解机制，“四张网”经验在全区推广。严厉打击各类违法犯罪，八类刑事案件破案率达到90%。我市荣获“全国社会治安综合治理优秀市”，青铜峡市荣获“全国平安建设先进县”，盐池县综治委荣获“全国社会治安综合治理先进集体”。食品药品安全、应急管理、防震减灾、外事、气象等工作取得新成绩。自觉接受人大法律监督、政协民主监督，办结人大代表议案建议73件、政协提案162件。全面推进依法行政，首部地方法规《吴忠市红色文化遗址保护条例》颁布实施，受到广泛好评。主动接受新闻舆论和社会公众监督，聚焦民生热点开展电视问政，广泛听取民主党派、工商联、无党派人士意见建议。支持工会、共青团、妇联等人民团体创新开展工作。严格落实中央八项规定精神，坚持不懈整治“四风”，三公经费下降19.93%。加强效能监察和审计监督，严肃查处“三不为”行为，问责干部121名，大力开展涉农扶贫领域腐败问题专项整治行动，始终保持惩治腐败高压态势，廉洁自律意识不断增强，工作作风持续好转，政府效能明显提升。

各位代表，2017年的成绩来之不易、令人振奋。这是习近平新时代中国特色社会主义思想指导引领的结果，是自治区党委、政府正确领导的结果，是市委总揽全局、科学谋划的结果，是人大、政协有力监督、大力支持的结果，是全市人民攻坚克难、奋力拼搏的结果。在此，我代表市人民政府，向奋战在各条战线的广大干部群众，向人大代表、政协委员、各民主党派、工商联，向离退休老同志和社会各界人士，向驻吴人民解放军、武警官兵、政法干警，向中央及区属驻吴单位，向关心支持吴忠发展的国家部委、自治区厅局、友好城市、政府顾问和外商、港商、台商表示崇高的敬意和衷心的感谢！

我们也清醒地认识到，工作上还存在许多不足，发展中也还有不少困难问题。一是综合实力不强，基础还不稳固，产业层次较低，转型升级步伐还需加快，发展不充分的问题依然突出。二是山川、城乡差距较大，基础设施建设和基本公共服务相对滞后，均等化明显不足，民生领域还有不少短板，脱贫攻坚任务艰巨，脱贫摘帽压力较大，发展不平衡的问题依然存在。三是科技创新能力不强，科技投入相对不足，创新人才短缺，创新氛围还不够浓厚。四是财政收支矛盾加剧，政府债务负担一直较重，保障性支出增大，一些涉及群众切身利益的问题还需下大力气解决。五是政府职能转变需要继续加快，作风建设还需持久用力，等等。我们将高度重视这些问题，认真深入研究，积极采取措施，切实加以解决。

2018年预期目标

各位代表，2018年是全面贯彻落实党的十九大精神的开局之年，是改革开放40周年，是决胜全面建成小康社会、实施“十三五”规划承上启下的关键一年，也是自治区成立60周年和地级吴忠市设立20周年，做好今年工作意义十分重大。当前，吴忠正处在爬坡追赶的关键时期，发展不足、粗放发展仍然是最大市情。党的十九大提出要加大力度支持革命老区、民族地区、贫困地区加快发展，强化举措推进西部大开发形成新格局，自治区第十二次党代会提出打造“两个先行区、两个示范区”、建设银川都市圈和沿黄生态经济带等战略部署，这为我市加快发展带来了新的重大机遇。站在这样一个新的历史方位，我们要准确把握新时代的新变化新要求，抓住用好机遇，积极应对挑战，对标要求、找准路径、聚焦发力，全力推动经济社会持续健康发展。

总体要求：以习近平新时代中国特色社会主义思想为指导，深入学习贯彻党的十九大精神，认真贯彻落实自治区第十二次党代会、十二届三次全会和市委五届五次全会精神，坚持稳中求进工作总基调，坚持新发展理念，坚持高质量发展，以创建全国文明城市等为载体，紧盯目标、坚守底线、发挥优势、补齐短板，统筹推进改革发展稳定各项工作，打好防范化解重大风险、精准脱贫、污染防治“三大攻坚战”，在打造“两个先行区、两个示范区”上干在实处、走在前列，为建设绿色经济示范市、决胜全面建成小康社会奠定坚实基础。

预期目标：地区生产总值增长8%，全社会固定资产投资增长8%左右，一般公共预算收入增长8%，社会消费品零售总额增长9%，城镇居民人均可支配收入增长8%，农村居民人均可支配收入增长9%，居民消费价格总水平涨幅控制在3%以内，城镇登记失业率控制在4%以内。完成自治区下达的环境质量改善和节能减排任务。

2018年，要在六个方面取得新进展、实现新突破。

推动高质量发展。坚持以供给侧结构性改革为主线，继续在“稳”上大力巩固，重点在“进”上积极突破，着力在“好”上聚焦发力，不断提高资本效率、土地效率、资源效率、环境效率，推动发展方式转变、经济结构优化、增长动力转换。

打好“三大攻坚战”。打好防范化解重大风险攻坚战，妥善化解政府债务问题，严厉打击金融违法违规行为，完善风险防控机制。打好精准脱贫攻坚战，聚焦深度贫困地区和盐同红交界处集中连片贫困地区，持续精准发力，巩固扶贫成果，提高脱贫质量。打好污染防治攻坚战，下大力气解决大气、水、土壤等环境突出问题，全力改善生态环境质量。

落实“三大战略”。创新驱动、脱贫富民、生态立区“三大战略”是自治区确定的重大战略。要把实施“三大战略”作为引领发展的重要抓手，全面落实“创新驱动30条”“脱贫富民36条”“生态立区28条”，认识上目标明确，行动上落实坚决，实现区域经济换道超车。

建设“一圈”“一带”。积极主动融入银川都市圈，推动产业发展协作互补、基础设施互联互通、公共服务共建共享。以绿色发展为引领，全力打造生态优先、绿色发展、产城融合、人水和谐的沿黄生态经济带。

促进乡村振兴。坚持农业农村优先发展，大力实施乡村振兴战略，按照产业兴旺、生态宜居、乡风文明、治理有效、生活富裕的总要求，健全城乡融合发展体制机制，推进农业供给侧结构性改革，推动农业全面提升、农村全面进步、农民全面发展。

补上发展短板。补上服务业发展短板，着力培育新增长点，形成新动能。补上民生短板，把人民对美好生活的向往作为最高奋斗目标，千方百计增加城乡居民收入，切实解决群众教育、就业、收入、社保、医疗、养老、居住、环境等方面的困难和问题，努力让全市人民生活更加幸福美满。补上全面建成小康社会短板，对标全面建成小康社会各项指标，突出抓重点、强弱项，确保各项目标如期实现。

今年重点任务

各位代表，今年的任务繁重艰巨，全市上下要进一步振奋精神、鼓足干劲、脚踏实地、埋头苦干，发扬钉钉子精神，各项工作要一件一件地用力抓，一项一项地去落实。

一、以项目建设为抓手，增强投资有效拉动

充分发挥项目建设对经济发展的关键作用，抓项目、增投资，推动经济平稳较快增长。

加快重点项目建设。加快哈纳斯500万吨天然液化气等200个重点项目建设，完成投资418亿元。实施伊利乳业产业园等118个产业项目，完成投资340亿元。落实棚户区改造、特色小城镇建设等35个基础设施项目，完成投资33亿元。抓好东南部引提调水及河道生态治理、盐同红革命老区脱贫引水工程等19个农林水项目，完成投资23亿元。建设太阳山至红寺堡公路、城东客运枢纽场站等12个交通运输项目，完成投资11亿元。加快同心、红寺堡职业教育中心等16个社会事业项目，完成投资11亿元。做实做细项目各项前期工作，加强要素保障，协调解决困难问题，力争4月份430个新建项目开工60%以上，237个续建项目全部复工，151个预备项目8月底前开工，46个重点前期项目取得实质性进展，确保项目前后接续、梯次跟进。

积极开展招商引资。突出精准招商、产业招商、以商招商，深化与京津冀、长三角、珠三角等重点区域的产业合作，围绕装备制造、农副产品加工、新能源等优势特色产业，引进一批科技含量高、市场潜力大、核心竞争力强的企业和项目，确保年内引进10亿元以上项目10个，20亿元以上项目5个，500强企业2家以上，招商引资到位资金增长10%。积极做好招商项目落地建设全过程服务，提高招商项目落地率。全力争项目引资金，确保上争项目资金不低于152亿元。

不断优化投资结构。提高产业投资和民间投资比重，拓宽项目融资渠道，积极推广PPP项目合作模式，引导支持民间资本进入基础设施、社会事业等领域，力争“两沟”水生态治理、黄河金岸文化旅游带等PPP项目列为国家、自治区示范项目。严格规范项目运作，把好资金投向，防止重复建设，提高投资质量效益。

二、以转型升级为核心，推动经济高质量发展

坚持质量第一、效益优先，促进一二三产业全面提质增效升级，加快构建产业特色鲜明、比较优势突出、市场竞争力强的现代化经济体系。

推进工业提质扩量。实施5000万元以上重点工业项目100个，力争中科能源、锦绣轻合金等50个项目达产达效，规上工业增加值增长9%以上。全力申报创建“中国制造2025”国家级示范区，充分发挥“中国制造2025”产业基金杠杆作用，加快发展装备制造、现代纺织等产业。加快建设中国自动化绿色精密铸锻产业园，全力打造装备制造产业聚集区。实施现代纺织业提升行动计划，建设恒成仁和年产15万锭纺纱等项目，进一步延伸上下游产业链。大力培育生物医药产业，开工建设太阳山医药科技（苏宁合作）产业园。深入开展“个转企”“小升规”行动，支持中小企业向“专精特新”方向发展，新增规上企业20户、亿元企业10户。坚决淘汰水泥、电石、铁合金等落后产能，实施青铝电解槽技改等25个重点技改项目，全面完成自治区下达的节能“双控”任务。积极整合优化工业园区，不断提升园区发展水平。实施金积工业园区一般固体废物存储处理等16个低成本化改造项目，实现园区污水处理全覆盖。加大自治区“降成本30条”等政策争取力度，帮助企业降本增效。

推进农业提档增效。坚持不懈稳基地、创品牌、拓市场，加快构建现代农业产业体系、生产体系和经营体系，农业增加值增长5%左右。粮食总产量稳定在90万吨，优质粮食种植面积稳定在100万亩。加快发展草畜、酿酒葡萄、枸杞等特色养殖种植，新增蔬菜瓜果20万亩。积极打造吴忠国家级奶产业园，建设宁夏奶牛研究院。推进利通区、青铜峡市“奶牛大县”，盐池县“滩羊大县”，同心县、红寺堡区“肉牛大县”建设，新增奶牛2万头、肉牛5万头、滩羊20万只、黑毛驴1万头。分类制定产业规划，组建酿酒葡萄、蔬菜等产业联盟。培育壮大新型经营主体，新增产业化重点龙头企业10家、市级以上示范性经营主体25个。鼓励发展休闲农业，打造法福来水稻景观、龙二葡萄长廊等田园综合体。吴忠国家农业科技园区创建为国家级生态农业有机区、现代农业休闲旅游区。提高农田水利基础设施建设质量，发展高效节水农业8万亩、改造盐碱地3万亩。加强防灾减灾、气象等工作，增强农业抗风险能力。

推进服务业提速升级。加快发展现代服务业，力争第三产业增加值增长9%。实施消费拉动经济增长行动计划，加快培育健康养老、休闲娱乐等新领域消费。加快创建国家电子商务示范市，年内实现电商覆盖90%以上的乡村。大力发展健康产业，积极培育中医中药、养生保健等新业态。加快老龄事业和产业发展，探索公建民营、医养结合等养老服务模式，逐步完善养老服务体系。大力发展会展经济，举办全国青少年航空航天模型锦标赛、盐池航空嘉年华及房车露营等30余项节会活动。推动万达广场、宁夏德昌铁路物流中心等商贸物流项目建成运营，改造提升太阳山镇等4个商贸集镇。做大做强吴忠“早茶经济”，打造“休闲商旅、食在吴忠”品牌。加快发展全域旅游，重点实施利通区城南生态文化公园、罗山东麓葡萄酒特色小镇等旅游项目，力促青铜峡黄河大峡谷•中华黄河坛、盐州古城历史文化旅游区创建为5A级景区。精心打造文化遗址、历史名城、节会庆典等旅游项目，提升“食、行、住、游、购、娱”全要素服务水平，接待游客和旅游收入分别增长12%和15%以上。

三、以创新驱动为引领，增强经济发展持续动力

加快创新驱动发展步伐，进一步提升产业和企业自主创新能力，打造创新型经济体系。

强化科技创新基础。认真落实创新驱动行动计划，鼓励企业加大研发投入，建立科技研发投入财政稳定增长长效机制，市县财政科技研发投入增长30%以上，带动全社会科技研发投入强度达到1.3%以上。探索实行科技创新券，建立灵活实用的科技创新扶持制度。以沿黄科技创新改革试验区建设为重点，围绕高端智能控制阀、滚动轴承、精密汽车零部件、现代纺织等产业，统筹推进各类创新平台和技术服务平台建设，建成各级各类创新平台10个以上。巧借“科技支宁”东风，深化与中国农科院、杨凌农业高新技术示范区、西北农林科技大学的交流合作，加快推进吴忠国家农业科技园区创建国家农业高新技术园区，建设宁夏（吴忠）富硒功能农业博士工作站。加大农业科技推广力度，农业科技贡献率达到60%以上。弘扬科学精神，提高全民科学素养，建成吴忠科技馆。

推动企业创新发展。鼓励建立以企业为主体、市场为导向、产学研深度融合的技术创新体系，引导支持企业加快创新。承办宁夏第五届创新创业大赛。培育国家级高新技术企业3家，科技型中小企业20家，新增自治区级企业技术中心1家。加快“两化”深度融合，新增全国两化融合贯标试点企业1家，全市规上企业数字化研发设计工具普及率达到35%以上。支持吴忠仪表建设严酷工况流体控制国家重点实验室和自治区级产业技术协同创新中心。实施人才引进和培养激励办法，让各类人才在创新中实现价值、创业中成就梦想。深入实施企业家素质提升行动计划，培育工匠精神，切实壮大本土创新人才队伍。

落实质量品牌战略。开展吴忠质量奖评选，促进产品质量提升。积极探索专利运营模式，争取在国家知识产权运营平台上设立吴忠运营窗口。开展知识产权贯标行动，新增发明专利50件以上，扩大企业专利优势。建立健全农产品生产标准化和质量安全监管体系，启动建设农产品追溯体系示范项目。加快推进农业“三品一标”认证，新增无公害农产品15个，绿色食品10个，地理标志农产品2个。做大做强“吴忠苹果”“盐池滩羊”“青铜峡大米”等地理标志品牌，积极创建“吴忠亚麻籽之乡”。全力打响“塞上硒都”品牌，制定富硒产品标准，新建富硒农产品基地23个、富硒农产品品牌10个，种植面积达到15万亩。新增中国驰名商标1个、宁夏名牌产品10个。

四、以脱贫富民为中心，切实保障改善民生

坚持尽力而为、量力而行，健全完善公共服务体系，办好10项30件民生实事，不断满足人民群众对美好生活的向往需要。

扎实推进精准脱贫。完成23个贫困村、2.7万人脱贫任务，统筹做好184个已脱贫销号村的巩固提升工作。实施“十三项脱贫行动计划”，全力加快红寺堡现代纺织扶贫产业园等7个盐同红交界处集中连片地区扶贫项目建设。加快道路交通和公共服务等基础设施建设，切实改善贫困地区生产生活条件和居住环境。努力提高贫困群众收入，大力发展滩羊、黑毛驴等特色产业，提升盐池黄花、同心中药材、红寺堡酿酒葡萄等种植基地建设水平，推动特色种养业规模化、集约化、标准化、市场化发展。大力发展资产收益扶贫项目，推进光伏、旅游、电商等扶贫新业态。加大金融扶贫力度，用活扶贫产业担保基金，力争贫困户扶贫小额信贷余额达到20亿元以上，贷款覆盖率达到73%以上。加大职业技能、实用技术、致富带头人培训，培训贫困人口1.5万人次以上。实行最严格的脱贫攻坚责任制，坚决杜绝急功近利、弄虚作假，确保扶贫工作务实，脱贫过程扎实，脱贫结果真实。

提高社会保障水平。实施积极的就业创业政策，推动稳定就业和更高质量就业，加大公益性岗位开发购买力度，新增就业1.4万人，实现农村劳动力转移就业20万人。推动企业建立以一线职工为重点的工资增长机制。深入实施全民参保登记计划，完善统一的城乡居民基本医疗保险制度和大病保险制度。全面实施医疗保险省级统筹，实现跨省异地就医住院直接结算。大力发展残疾人事业，提高残疾康复服务水平。强化低保等社会救助动态管理，落实城乡低保标准自然增长机制，织牢特困群众生活保障网。

全面发展社会事业。坚持教育优先发展，加快义务教育学校标准化建设，推进东片区初级中学等项目建设，打造标准化学校示范样板。实行优质教育资源集团化办学，深化与银川一中等9所学校的合作，组建利通一小、吴忠中学等18个教育均衡发展协作体，有效解决中小学生课外负担重、“择校热”“大班额”等问题。完善现代职业教育体系，推动职业教育特色化发展、品牌化打造。全面加快健康吴忠建设，推进优质医疗资源下沉，进一步优化基层公共卫生服务，构建现代医疗卫生体系。积极与自治区人民医院、宁夏医科大学附属医院、自治区妇幼保健院3个医疗集团开展合作，提升医院综合服务能力。在盐同红交界处设立县级人民医院分院，解决群众看病远、看病难问题。深入实施文化惠民工程，广泛开展送戏下乡、公益放映和全民阅读活动，发展壮大文化产业，建成吴忠美术馆，成功创建为第三批国家公共文化服务体系示范区。以创建全国文明城市为总揽，深入开展国家低碳城市试点等9个“国字号”创建。高标准承办好自治区第十五届运动会和第七届宁夏黄河金岸（吴忠）国际马拉松等体育赛事。

五、以绿色发展为统领，推进生态立市进程

加快推进环境保护和生态文明建设，坚持全民共治、源头防治、综合治理，让绿色发展深入人心、绿色财富持续增加、绿色生活成为自觉，还自然以宁静、和谐、美丽。

推进植绿护绿。全力创建国家森林城市，实施大规模国土绿化行动，完成营造林30万亩，全市森林覆盖率达到16.5%。新建改造城市绿地1860亩，进一步扩大绿地面积、提升绿化品位。科学划定林地和森林、湿地、荒漠植被、物种保护四条生态保护红线，严格落实森林资源保护发展责任制。推进自然保护区规范化建设和监管，筑牢以罗山、哈巴湖、青铜峡库区为重点的生态安全屏障。加强野生动物保护，深化禁牧封育成果。

防治大气污染。强化源头控制，综合施策，严管重罚，力争空气质量优良天数比例达到80%。实施水泥、火电等重点行业提标改造工程，大幅降低污染物排放强度。加大燃煤锅炉淘汰力度，提高清洁能源利用比例，城市建成区内35蒸吨以下燃煤锅炉全部淘汰。严格落实扬尘污染“6个100%”防控措施，从严整治工业堆场、建筑施工扬尘污染。加强机动车污染防治，加快淘汰黄标车。严禁秸秆焚烧，拓宽农作物秸秆综合利用渠道，年内综合利用率达到89%。加强重污染天气应对处置，利通区、青铜峡市实行联防联控。

防治水体污染。严格落实河长制，启动实施湖长制，推动治水常态化长效化。加快实施清水沟、南干沟水环境综合治理项目，建设吴忠第四、第五污水处理厂，启动吴忠第一污水处理厂、青铜峡新材料基地污水处理厂等人工湿地项目，全力确保入黄排水沟水质达到_x0007_IV类、黄河断面水质稳定在_x0007_II类优标准。启动实施盐池县狼布掌等12个小流域综合治理工程，治理水土流失面积240平方公里。实施清水沟等水系连通工程，打造水润吴忠生态水系。建成新水源地，加强水源地保护。深入推进区域供水和农村饮水安全工程，加快盐池县、同心县脱贫饮水配套工程和红寺堡区农村饮水工程建设。

防治土壤污染。加大农用地保护力度，划定农用地土壤环境质量类别。降低化肥、农药使用量，鼓励残膜、塑料、粪污综合化利用，切实减少农业面源污染。开展土壤污染状况调查，建立全市土壤环境基础数据库和土壤环境信息化管理平台。加强涉危企业规范化管理，建立非法处置危险废物企业黑名单制度，严格危险废弃物处置和利用。

六、以区域协调为契机，主动融入银川都市圈

认真落实《吴忠市空间规划》，加强空间治理和管控，扎实推进城乡融合发展。加快利青同城化进程，积极融入银川都市圈，建设银川都市圈靓丽南翼。

构建互联互融交通。配合实施好银西高铁、城际铁路、京藏高速公路改扩建、银百银昆高速等重大交通项目。开通黄河3公里旅游航线，启动建设黄河吴忠段航运二期项目，实现局部通航。加快推进青铜峡至银川正源街快速通道建设，开工建设吴忠黄河公路大桥与滨河大道连接线等项目，完成国道344线吴忠至灵武段公路建设。落实农村公路“四好”要求，新建农村公路400公里。

完善城市服务功能。全面开展城市“双修”，实施市区夜景亮化、交通路口改造等工程，着力提升城市品位。加快建设银西高铁枢纽站片区6平方公里生态商业街区，优化城市北片区服务功能。新建、续建铁西路、朝阳东街等10条城市道路，延伸地下综合管廊4.2公里，实施棚户区改造7527套。装配式建筑比例达到5%以上。强化城市交通管理，建设完善智慧公交系统，着力解决交通拥堵、停车难等“城市病”。

加快建设美丽乡村。围绕一村一貌、一镇一品，挖掘文化内涵，发展特色产业，提升公共服务水平，培育一批功能完善、特色彰显、环境优美、宜居宜业的产业重镇、风情小镇、旅游名镇。实施韦州镇、大水坑镇、金银滩镇等5个特色小镇项目，改造危窑危房3000户以上，建设美丽村庄20个。大力推进“厕所革命”，实施农村改厕4000户以上。深入实施农村环境综合治理行动计划，制定城乡环境卫生治理地方法规，建立完善长效管理机制，进一步改善农村人居环境。

七、以改革开放为动能，充分释放发展新活力

按照市场化、便利化原则，进一步深化改革，以改革思维和开放方式破解瓶颈、激发活力、推动发展。

推进重点领域改革。加大“放管服”改革力度，全面落实“两个清单”动态调整机制。加快“多证合一”改革，探索开展个体工商户简易登记。积极推进“不见面、马上办”审批服务模式，力争不见面办理事项达到80%。规范国有企业运营，完善现代企业管理制度，推进国有企业混合所有制改革，发挥投融资平台作用，做强做优做大国有资本。深化医药卫生体制改革，坚持“三医联动”，启动市县公立医院医疗服务价格调整工作，公立医院药占比控制在30%以下。建成运行吴忠农村产权交易中心，引导规范土地经营权有序流转，探索土地入股、土地托管等经营方式。全面开展农业水价综合改革，积极推进水权交易，实现水资源高效利用。开征环保税和水资源税，形成有效约束激励机制。

防范化解重大风险。严格执行《预算法》，加快构建科学、规范、透明的财政管理体系。不断强化审计监督，规范债务管理，严格控制债务余额规模和增长速度，妥善化解政府债务问题。严厉打击金融违法违规行为，完善风险防控机制，确保金融安全。“坚持房子是用来住的，不是用来炒的”，严格控制增量，逐步消化存量，保持房地产市场平稳健康发展。

扩大对外开放合作。深入实施开放吴忠“十大行动”，巩固提升35个吴忠优质特色产品展示展销中心运营管理水平，组建产销联盟，力争年度销售额突破3亿元。积极对接银川综合保税区、空港口岸，推动企业贸易服务便利化。全力支持出口企业设立境外销售窗口，发展跨境电商，全年进出口总额增长10%以上。积极引入银行、证券、保险等金融机构，力争新设金融分支机构2家。

八、以法治建设为重点，保持社会大局和谐稳定

加强和创新社会治理，巩固发展政通人和、安定有序的良好局面。

大力推进依法治市。把法治作为未来发展的核心竞争力，大力实施法治吴忠建设“五大行动计划”。严格执行重大行政决策程序规定，健全重大决策合法性、风险性和廉洁性审查评估机制。加快构建决策科学、执行坚决、监督有力的权力运行体系，落实行政执法责任制，推进规范文明执法。加快“法律八进”示范点创建活动，落实“七五”普法责任制。健全人民调解、行政调解、司法调解“三位一体”调处机制，依法规范信访秩序。加强诚信体系建设，建立完善诚信激励机制和失信惩戒机制，进一步提高社会信用水平。

加强民族团结进步。大力开展“民族宗教工作创新提升年”活动，全面加强和改进新形势下民族宗教工作，着力巩固民族团结、宗教和顺的社会环境。全面贯彻党的民族政策，加强各民族交往交流交融，深化“585”创建模式，持续推进民族团结进步创建“三个十”行动，巩固提升民族团结进步创建成果。坚持宗教中国化方向，依法依规管理宗教事务，依法打击宗教领域非法渗透活动，积极引导宗教与社会主义社会相适应。

加强和创新社会治理。深化“平安吴忠”建设，全面提升基层社会治理和社区便民服务水平。大力推进社会治安防控“六张网”建设，依法严厉打击各种违法犯罪活动，深入推进禁毒人民战争。严格落实安全生产责任制，加快推进安全生产领域改革，努力提升安全监管水平。健全食品药品安全监管网络和执法体系，加快建设食用农产品快检室，建成市食品检验检测中心。

九、以服务人民为宗旨，改进政府工作作风

中国特色社会主义进入新时代，新的形势和任务对政府工作提出了新的更高要求。我们要加快转变政府职能，切实改进作风，提高行政效能，做到信念过硬、政治过硬、责任过硬、能力过硬、作风过硬，不断增强政府执行力、公信力和服务力。

始终坚持党的领导。深入学习领会习近平新时代中国特色社会主义思想，牢固树立政治意识、大局意识、核心意识、看齐意识，始终同以习近平同志为核心的党中央保持高度一致，切实把思想和行动统一到党的十九大精神上来。严格遵守党的政治纪律和政治规矩，自觉向党中央看齐，向党的理论和路线方针政策看齐。全面贯彻落实党中央、国务院和自治区各项决策部署，紧紧围绕市委确定的战略、目标、任务狠抓落实。自觉接受市人大及其常委会的法律监督和市政协的民主监督，认真办理人大代表建议和政协委员提案。主动接受社会监督和舆论监督。扎实开展“不忘初心、牢记使命”主题教育。坚持从严管理，不断加强政府及组成部门党组政治建设、思想建设、组织建设、作风建设和纪律建设。

始终践行勤政为民。主动加强学习，注重调查研究，开阔思路视野，找准短板弱项，解决实际问题，提高推动改革发展能力。坚持务实勤政，大力倡导说了就办、定了就干、干就干好。优化绩效考核和督查问责，落实正向激励和容错纠错机制，坚决整治不作为、慢作为、乱作为。加大政务公开和政府信息公开力度，提高政府工作透明度。

始终做到清正廉洁。贯彻全面从严治党要求，严格落实党风廉政建设“一岗双责”和领导干部廉洁从政各项规定，始终把纪律和规矩挺在前面。加强重点领域环节监管，严控“三公”经费，严查重点领域、关键岗位和扶贫领域腐败行为。严格落实中央八项规定精神和自治区、市委有关要求，坚决纠正“四风”，持之以恒正风肃纪。

各位代表！新时代要有新作为，新的一年要有新气象。我们深知，全市人民对吴忠发展充满期盼，对美好生活满怀向往。我们深感，幸福都是奋斗出来的，必须踏踏实实、一步一个脚印。让我们以习近平新时代中国特色社会主义思想为指导，全面贯彻落实党的十九大和自治区第十二次党代会精神，在市委的坚强领导下，不忘初心、牢记使命，干在实处、走在前列，推动吴忠经济社会创新发展、协调发展、绿色发展、开放发展、共享发展，为谱写全面建成小康社会新篇章、开创新时代吴忠发展新局面而努力奋斗！
